# Temporal Patterns of Diversification across Global Cichlid Biodiversity (Acanthomorpha: Cichlidae)

**DOI:** 10.1371/journal.pone.0071162

**Published:** 2013-08-19

**Authors:** Caleb D. McMahan, Prosanta Chakrabarty, John S. Sparks, Wm. Leo Smith, Matthew P. Davis

**Affiliations:** 1 LSU Museum of Natural Science (Ichthyology), Department of Biological Sciences, Louisiana State University, Baton Rouge, Louisiana, United States of America; 2 American Museum of Natural History, Department of Ichthyology, Division of Vertebrate Zoology, New York, New York, United States of America; 3 The Field Museum, Division of Fishes, Chicago, Illinois, United States of America; Aberystwyth University, United Kingdom

## Abstract

The contrasting distribution of species diversity across the major lineages of cichlids makes them an ideal group for investigating macroevolutionary processes. In this study, we investigate whether different rates of diversification may explain the disparity in species richness across cichlid lineages globally. We present the most taxonomically robust time-calibrated hypothesis of cichlid evolutionary relationships to date. We then utilize this temporal framework to investigate whether both species-rich and depauperate lineages are associated with rapid shifts in diversification rates and if exceptional species richness can be explained by clade age alone. A single significant rapid rate shift increase is detected within the evolutionary history of the African subfamily Pseudocrenilabrinae, which includes the haplochromins of the East African Great Lakes. Several lineages from the subfamilies Pseudocrenilabrinae (Australotilapiini, Oreochromini) and Cichlinae (Heroini) exhibit exceptional species richness given their clade age, a net rate of diversification, and relative rates of extinction, indicating that clade age alone is not a sufficient explanation for their increased diversity. Our results indicate that the Neotropical Cichlinae includes lineages that have not experienced a significant rapid burst in diversification when compared to certain African lineages (rift lake). Neotropical cichlids have remained comparatively understudied with regard to macroevolutionary patterns relative to African lineages, and our results indicate that of Neotropical lineages, the tribe Heroini may have an elevated rate of diversification in contrast to other Neotropical cichlids. These findings provide insight into our understanding of the diversification patterns across taxonomically disparate lineages in this diverse clade of freshwater fishes and one of the most species-rich families of vertebrates.

## Introduction

Recent studies that focused on groups long considered to be the product of rapid evolution (e.g., skinks, perch-like fishes, passerine birds) have demonstrated that these lineages have undergone periods of increased diversification in their evolutionary history that may explain their exceptional present-day diversity (e.g. [1], [2], [3]). Cichlids have often been regarded as a lineage that exhibits elevated diversification rates in comparison to other freshwater-fish lineages (e.g. [4], [5], [6], [7]), and these elevated diversification rates are often associated with purported “adaptive radiations” (e.g. [4], [5], [6], [8]). However, a robust temporal phylogenetic hypothesis for the family Cichlidae, comprising a broad taxonomic sampling across all the major worldwide lineages that would permit investigators to examine why some cichlid lineages within a geographic assemblage are depauperate (e.g. oscars, angelfishes, jewel cichlids), whereas others are notably species rich (e.g. African rift-lake cichlids), is currently lacking.

Cichlids are among the largest lineages of freshwater fishes, with more than 1,600 valid species [9], [10]. It has been hypothesized that this incredible diversity is often associated with increased diversification rates due to the exploitation of novel habitats and environments [7], with high levels of morphological disparity correlated with ecological niches. Hybridization has also possibly acted as an aid to diversification in these fishes [11], [12]. Groups such as the haplochromin cichlids of Lakes Victoria and Malawi, known for their colorful species flocks [13], [14], are considered to be the product of adaptive radiations. Therefore, they have been thought to have evolved with an increased diversification rate relative to other cichlid lineages [6]. However, as noted by Alfaro et al. [2], it is possible that some “classical” examples of exceptional radiations may not truly be so exceptional. For instance, the low species richness of the non-haplochromin African cichlids relative to haplochromins could be the result of a diversification rate shift decrease, rather than a rate shift increase in the haplochromins. A comparative study of cichlid diversification across all major lineages is required to tease apart the patterns of diversification that have shaped present day cichlid diversity.

The bulk of cichlid evolutionary studies have focused on the East African Great Lake cichlids (e.g. [4], [6], [13], [15]), with an emphasis on the exceptional morphological disparity of these cichlids and their ecological niches [16], [17], [18]. Day et al. [6] investigated diversification rates of African rift-lakes cichlids and suggested that Lake Tanganyikan lineages have diversified at a slower rate than those in lakes Malawi and Victoria. Despite the fact that there are over 500 species, relatively few studies have investigated diversification rates in Neotropical cichlids, with only two tribes being the focus of prior study. Hulsey et al. [7] investigated the accumulation of heroin lineages through time and found no evidence for an early burst of speciation in the group; instead, a continuous pattern of diversification through time was shown. Their results indicate that the diversification of heroin cichlids has not slowed over time, neither due to processes associated with density-dependent speciation, nor a decrease in diversification rate [19]. The radiation of the Neotropical geophagins (eartheaters) was hypothesized to represent an adaptive radiation by López-Fernández et al. [20], [21], based solely on their short branch lengths among geophagin lineages and the overall diversity of ecomorphological specializations across the group. Short basal branch lengths were also used to propose early bursts of divergence in the Heroini [21]. Later, López-Fernández et al. [22] used lineage through time plots to indicate that Neotropical cichlids, particularly the geophagins, show signatures of early bursts of diversification (density-dependent). They conclude that early radiations of the geophagin cichlids likely affected or limited the diversification of other Neotropical cichlid clades [22].

**Figure 1:**
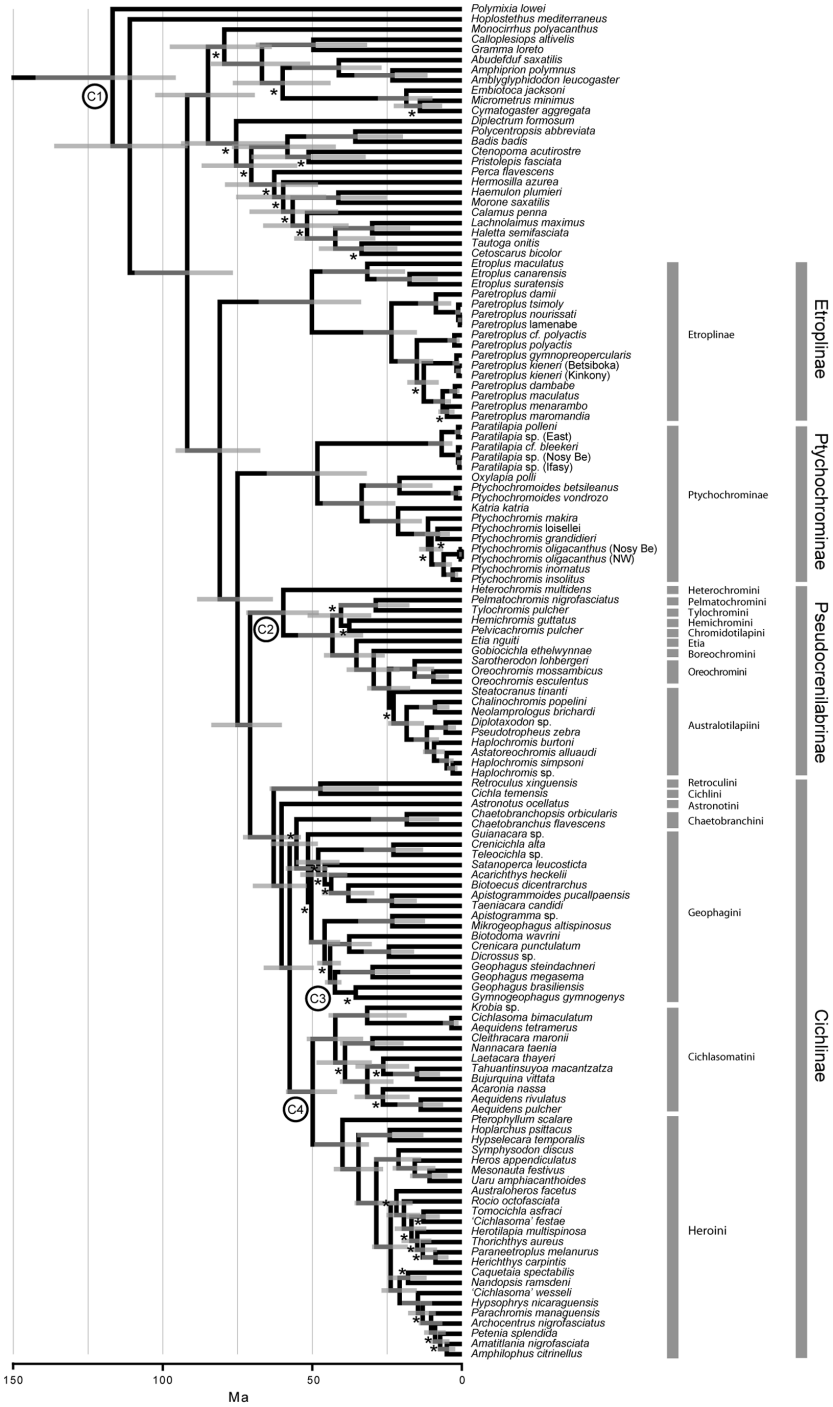
Temporal phylogeny of cichlid fishes based on two mitochondrial (16S, COI) and two nuclear genes (TMO, H3). C1 indicates Acanthomorpha calibration; C2 indicates †*Mahengechromis* calibration; C3 indicates †*Gymnogeophagus eocenicus* calibration; C4 indicates †*Plesioheros* and †*Trembichthys* calibration. Horizontal gray bars indicate age range of 95% HPD. * at nodes indicates BPP ≤95.

In addition to interest in their diversification, cichlids have been the focus of numerous biogeographic studies given their broad Gondwanan distribution [23], [24]. The family Cichlidae comprises four subfamilies (*sensu* Sparks and Smith [24]; Fig. 1): Etroplinae distributed in Madagascar, India, and Sri Lanka with 16 valid species; Ptychochrominae endemic to Madagascar with 15 valid species; the African, Iranian, and Middle Eastern Pseudocrenilabrinae with 1081 valid species; and the entirely Neotropical Cichlinae with 526 valid species. Previous divergence time studies that have included cichlids have recovered a wide range of potential divergence estimates for the family, and the age for the common ancestor of cichlids has only been explored in a handful of studies, few of which have utilized fossil cichlids as calibration points (e.g. [25], [26], [27]). Divergence estimates for Cichlidae in the study of Azuma et al. [26] range from the Early to Late Cretaceous (115–78 Ma) based on mitogenomic data and strictly Gondwanan fragmentation calibrations. Genner et al. [25] recovered drastically different ages for the Cichlidae, with an Early Cretaceous origin (133 Ma) based on geophysical calibrations, and an Eocene origin (46 Ma) based on available fossil calibrations. Murray [28] hypothesized that cichlids may have originated sometime during the Cenozoic; however, this supposition was based solely on the distribution of known cichlid fossils at that time. Presently, there are no robust temporal hypotheses of cichlid divergence times that utilize the complete fossil record of cichlids (including the most recently discovered fossils) with a broad and comprehensive taxonomic sampling of all major geographic lineages.

The oldest known fossil cichlids from Africa (†*Mahengechromis*) are Eocene in age (approximately 46 Ma) from the Mahenge formation in Tanzania [28], [29], and these fossils are clearly members of the African subfamily Pseudocrenilabrinae [28]. A number of fossil cichlids are also known from South America, specifically from Brazil and Argentina, with specimens dating from the Miocene to the Eocene [30], [31], [32], [33]. A number of new fossil cichlid taxa from the Neotropics have recently been described that represent some of the oldest known cichlids, dating back to the Eocene (approximately 40–49 Ma, [32], [33]). The placement of these fossils has necessarily been based on morphological phylogenetic studies incorporating extant and extinct taxa [32], [33]. These recently discovered extinct taxa present an opportunity to utilize several novel fossil calibrations for investigating estimates of cichlid divergence times not available to previous researchers.

The contrasting distribution of species diversity across the major lineages of cichlids demands an investigation of whether different rates of diversification explain the disparity in species richness across these lineages. The purpose of this study is to investigate the tempo of diversification within and across the major lineages of cichlids, with an emphasis on the diversification patterns of Central and South American cichlids (subfamily Cichlinae) in the context of the entire cichlid radiation. We establish a robust temporal phylogeny of cichlids that includes broad taxonomic sampling of all major lineages (tribes) that, in turn, provides a framework for studying patterns of diversification across global cichlid biodiversity. We (1) investigate the phylogenetic history and temporal divergence of cichlids, (2) test for the presence of significant rate shifts (increases or decreases) in diversification across cichlid lineages, and (3) explore whether any cichlid lineages exhibit exceptional species richness given their estimated age of divergence.

## Methods

### Data Acquisition

Taxonomic sampling of the family Cichlidae included a representative of every genus from the subfamilies Etroplinae and Ptychochrominae (2 and 4, respectively), 17 genera from the subfamily Pseudocrenilabrinae (17/42), and 54 genera from the subfamily Cichlinae (54/57; representing all seven tribes). Sequence data from the previous phylogenetic and taxonomic studies of Cichlidae from Sparks and Smith [24] and Smith et al. [34] were used in this study because their works include the greatest global taxonomic coverage of the family Cichlidae to date. The dataset used here (Table S1) included two mitochondrial (large ribosomal subunit 16S, COI) and two nuclear (histone H3, Tmo-4C4) genes for a total of 2,069 aligned nucleotides. Outgroup sampling included a diversity of acanthomorph lineages from 19 families, including 17 perciform families (*Table* S1). Outgroup sampling was based on the phylogenetic hypothesis of Wainwright et al. [35].

### Phylogeny Reconstruction and Divergence Time Estimation

Sequences were aligned with MAFFT [36] using default parameters. All alignments were visually inspected and concatenated in MESQUITE v1.7 [37]. The sequence alignment is available in the Dryad Digital Repository (http://datadryad.org/). Topology reconstruction and relative divergence times were estimated simultaneously in BEAST v1.6.2 [38] using a template from BEAUTI v1.6.2 and a Yule speciation model, with results visualized in TRACER v.1.5 [39]. Each gene was assigned a separate model (COI and histone H3, GTR + I + G; 16S, GTR + G; Tmo-4C4, HKY + G), which was recommend by jMODELTEST [40] using the Akaike information criterion (AIC). Mean substitution rates were not fixed, and substitution rates were estimated under a relaxed uncorrelated lognormal clock that allows for independent rates to vary on different branches within the topology [41]. Under this model there is no *a priori* correlation between any rates in the tree. Fossil calibrations were assigned a lognormal prior, with hard minimum ages of clades set *a priori*. The minimum dates were assigned based on the oldest known fossil for each clade (Methods S1, Figure S1, Figure S2). In order to assess the phylogenetic placement of Neotropical cichlid fossils **†** *Plesioheros* and **†** *Tremembichthys*, we conducted a combined molecular and morphological genus-level analysis of Cichlinae (*Methods* S1, *Figure* S1, *Figure* S2, *Table* S2). Four separate analyses were performed with 50 million generations each, and a burn-in of 5 million generations for each analysis. Parameters and trees were sampled every 1,000 iterations for a total of 200,000 trees, 180,000 post burn-in. The program Tracer v 1.5 [39] was used to inspect the effective sample size (ESS) of all parameters in each analysis and to verify parameter stationarity. All parameters appeared to converge on a stationary distribution and possessed ESS values greater than 200, suggesting that all analyses satisfactorily sampled the posterior distributions of each parameter. A 50% maximum clade compatibility (mean heights) tree was generated from the posterior tree distribution and served as a framework for diversification analyses. Additionally, independent analyses were performed that sampled only from the prior in order to assess the impact the prior may have on the results, and we detected no evidence that the prior (without the data) directly impacted the evolutionary relationships indicated by our topological estimations.

### Diversification Rate Variation

The resulting maximum clade compatibility tree from BEAST (Fig. 1) was trimmed to exclude all non-cichlid taxa. Additionally, this tree was pruned further for use in the various diversification analyses described below. The first topology (Fig. 1) included one representative for each monophyletic subfamily as a terminal for use in combined taxonomic and phylogenetic analyses that included information regarding the known valid species diversity for each subfamily assigned to its respective terminal. The number of taxonomically valid species for each lineage was derived from the current number of valid species listed in *Catalog of Fishes* [42]. We included only valid, taxonomically recognized species in our estimates of lineage diversity. These estimates are discussed in Methods S1.

Models of diversification rate shifts were calculated using MEDUSA [2] in R, and implemented in the Ape [43] and GEIGER [44] libraries. The MEDUSA analysis estimates rates of speciation and extinction on a chronogram that incorporates taxonomic information. The pruned topologies (subfamily and tribe) with accompanying taxonomic information were utilized for this analysis. The maximum likelihood MEDUSA method begins by estimating birth and death values and an AIC score for a model with no shifts in diversification and a single birth and death value across the tree. The method then fits models of increasing complexity by incorporating a branch where rates of diversification change, with an additional birth and death value calculated for the clade where the shift point occurred. If the new model has an AIC score that is lower than the previous model by an AIC cutoff value determined by the researcher (4 is a common threshold for AIC significance and is recommended by Alfaro et al. [2]), then the model incorporating a rate shift is retained. This step-wise procedure continues adding additional shift points throughout the tree until the AIC threshold criterion is no longer met. At this point, a backward elimination procedure begins that individually removes shift points and reevaluates the models. After both a forward and downward step, a single model is chosen as the most likely [2].

We used the methodology of Magallón and Sanderson ([45]; eqn 8–11) as implemented in the R platform package GEIGER [44] to test whether cichlid subfamilies and tribes exhibit statistically significant higher or lower species richness given clade age. This method calculates a 95% confidence interval (CI) of the potential expected number of species within a clade given a net diversification rate (*r*), a relative extinction rate (*eps*), and clade age. A plot of CI ranges was generated for a net diversification rate calculated from an estimator of *r* implemented in GEIGER that incorporates taxonomic information with a temporal phylogeny. Ranges for the CI values were calculated for two separate *eps* values that represent possible low and high relative extinction rates (*eps*  = 0, 0.9). The estimated *r* was 0.0828 under a model of low relative extinction rates (*eps*  = 0), and 0.062 under a model of high relative extinction rates (*eps*  = 0.9). We also calculated CI ranges for the background rate of diversification and relative extinction rate indicated from the MEDUSA analysis (*r* = 0.069, *eps*  = 0.41). Clade age for each cichlid lineage was then plotted against the number of known valid species in that lineage within the context of the 95% CIs that were generated. Cichlid clade ages were based on the mean clade ages estimated from the BEAST analysis. If the known species diversity for a lineage given its age lies outside either the upper or lower CI bounds of expected taxonomic richness, then that clade is subject to statistically significantly high or low diversification.

## Results

**Figure 2:**
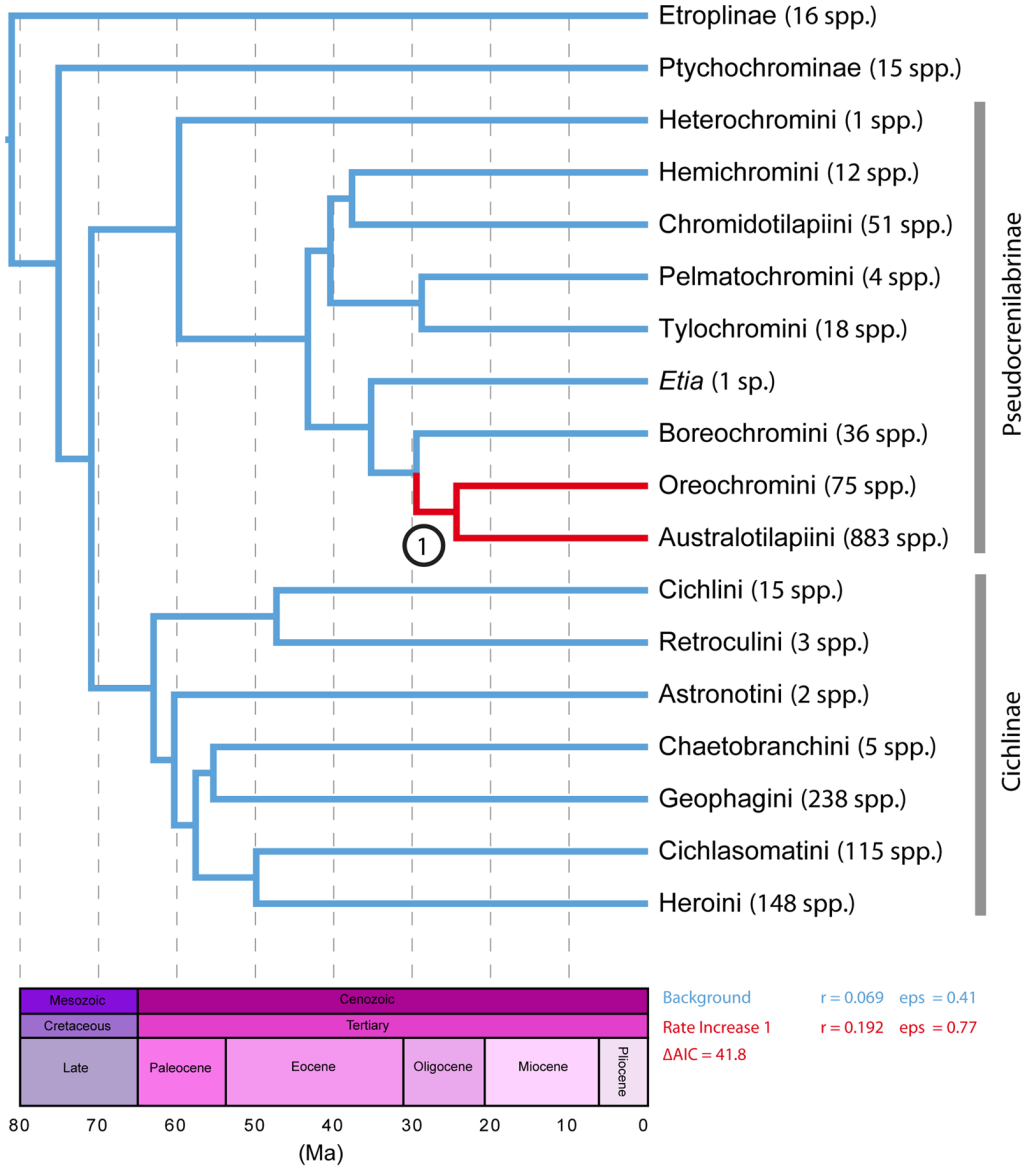
Temporal phylogeny of cichlids pruned to subfamily for Ptychochrominae, Etroplinae, tribes for Pseudocrenilabrinae, Cichlinae. Red clades indicate rate shifts in diversification, with lineages in blue undergoing a background rate of diversification.

The maximum-clade compatibility tree with 95% higher posterior densities (HPD) from our divergence time analysis of four gene fragments and 108 cichlid taxa across every major lineage is shown in Figure 1. The HPDs correspond to the 95% interval of age ranges sampled for each node in the posterior distribution. Posterior probabilities and HPD ranges for cichlid subfamilies and tribes (Fig. 1) are listed in Table 1. The family-level phylogeny recovered is consistent in relationships to those of Sparks and Smith [24] and Smith et al. [34]. The pruned tree (Fig. 2) summarizes the combined taxonomic and phylogenetic data for our diversification analyses.

**Table 1:** Divergence times of cichlid lineages, as seen in Figures 1 and 2.

Lineage	Mean Age (Ma)	95% HPD Age (Ma)
Cichlidae	81	67–96
Subfamily Etroplinae	50	34–68
Subfamily Ptychochrominae	48	32–65
Subfamily Pseudocrenilabrinae	60	48–72
Tribe Heterochromini	60	48–72
Tribes Hemichromini + Chromidotilapiini	38	30–52
Tribes Tylochromini + Pelmatochromini	29	17–41
*Etia*	35	25–46
Tribe Boreochromini	29	20–38
Tribe Oreochromini	16	9–23
Tribe Australotilapiini	23	17–31
Subfamily Cichlinae	63	54–74
Tribes Cichlini + Retroculini	47	28–64
Tribe Astronotini	60	52–70
Tribe Chaetobranchini	18	8–30
Tribe Geophagini	52	40–51
Tribe Cichlasomatini	42	33–52
Tribe Heroini	40	31–49

A monophyletic Cichlidae was recovered with strong statistical support (posterior probability  = 0.99) and an estimated divergence of the family in the Mesozoic, specifically during the Late Cretaceous (95% HPD 96–67 Ma, Fig. 1). The four cichlid subfamilies Etroplinae, Ptychochrominae, Pseudocrenilabrinae, and Cichlinae were recovered as monophyletic with strong statistical support and with estimated divergences largely during the Cenozoic, specifically in the Paleocene and Eocene (68–43 Ma; Figs. 1, 2, and Table 1). The major clades within the African subfamily Pseudocrenilabrinae and Neotropical subfamily Cichlinae were shown to have diverged between the Eocene and Miocene (Figs. 1, 2, and Table 1).

The ultrametric tree (Fig. 1) was pruned to include a representative of each of the subfamilies Etroplinae and Ptychochrominae, and each available tribe within the subfamilies Pseudocrenilabrinae and Cichlinae (Fig. 2). Species-richness numbers correspond with currently recognized valid species (e.g., [9], [10], [42]) and were matched to each terminal (Fig. 2) for analyses that included a combination of phylogenetic and taxonomic information.

We tested for shifts in diversification rates utilizing a maximum-likelihood approach that incorporates taxonomic and phylogenetic data (see Methods). The maximum-likelihood step-wise AIC model test methodology MEDUSA [2] indicates that there is strong evidence for a single net diversification rate shift (speed up) within Cichlidae when analyzed on the phylogeny that included representatives for the subfamilies Etroplinae and Ptychochrominae and representatives for tribes within subfamilies Pseudocrenilabrinae and Cichlinae (Table S1, Fig. 2). For a detailed discussion of the lineages examined and species richness within these subfamilies, see the Methods section (Table S1, Fig. 2). As shown in Figure 2, the MEDUSA analysis identified a five-parameter birth and death model with a single rate increase in the African Pseudocrenilabrinae, at the most recent common ancestor of the Oreochromini + Australotilapiini clade (AIC  = 294.8), as the best fit for these data when compared to the two parameter single birth and death model that indicates a constant diversification rate across cichlid lineages (AIC  = 336.6). The ΔAIC score between the rate constant and rate variable model is 41.8, greater than the significance cutoff of 4 suggested by Alfaro et al. [2], which indicates that the model incorporating a single rate shift fits the data significantly better than that which assumes a constant diversification rate. No significant shifts in diversification were detected within the other three cichlid subfamilies, comprising lineages found in Madagascar, India, South America, or Central America.

**Figure 3:**
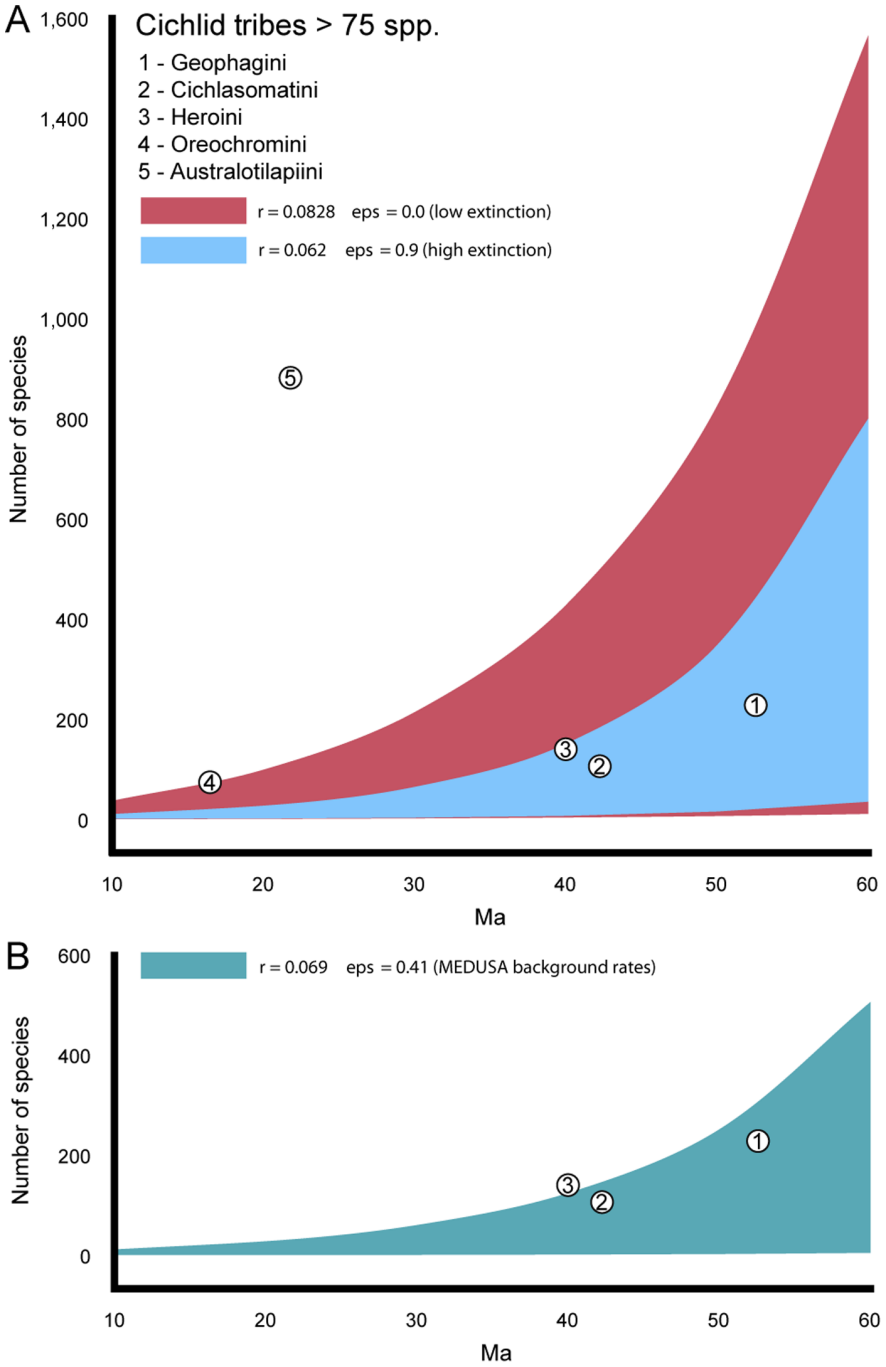
Clade age vs. species richness in cichlid tribes with greater than or equal to 75 species. Area curves indicate 95% confidence intervals for upper and lower bounds of species diversity given clade age for A; low (*r* = 0.0828, *eps*  = 0) and high (*r* = 0.062, *eps*  = 0.9) relative rates of extinction (*eps*) given a constant net rate of diversification (*r*) across cichlids, and B; the estimated background rate of diversification (*r* = 0.069) and relative rate of extinction (*eps*  = 0.41) from the MEDUSA analysis (Fig. 2). White circles indicate the mean clade age for each tribe from the temporal hypothesis of cichlid evolutionary relationships (Fig. 1). Lineages appearing to the left of the curves indicate exceptional species richness given clade age.

We used the likelihood methodology of Magallón and Sanderson [45] to calculate a 95% confidence interval (CI) for the expected number of species given time. This allows us to test whether cichlid subfamilies and tribes exhibit statistically significant high or low species richness if diversification rates were constant across the family (Fig. 3A) and incorporating the potential of multiple rates (Fig. 3B). The plot of 95% confidence intervals for expected species richness of a clade over time is shown in Figure 3. Confidence intervals were calculated under a relative diversification rate (*r*) estimated from the combined taxonomic information of known cichlid diversity and our temporal phylogenetic hypothesis with two relative rates of low (*eps  = *0, estimated *r* = 0.0828) and high (*eps*  = 0.9, estimated *r* = 0.062) extinction (Fig. 3A), and the estimated background rate from the MEDUSA analysis (Fig. 3B; *eps*  = 0.41, estimated *r* = 0.069). The taxonomic richness of highly diverse cichlid lineages, as shown in Figure 3A, indicates that only the African tribe Australotilapiini unambiguously falls outside the expected species richness CIs given clade age (23 Ma) when considering the HPD range of estimated divergence ages and rates of relative extinction. The tribe Oreochromini was also found to potentially exhibit exceptional species diversity given clade age (16 Ma); however, this result depends on the age of potential divergence and the relative rates of extinction (Fig. 3). Only three lineages from the subfamily Cichlinae are highly diverse, with over 75 species each and the potential for exhibiting exceptional species richness (Geophagini, Cichlasomatini, and Heroni). For the tribes Geophagini and Cichlasomatini, their exceptional species richness is potentially explained by clade age alone (52 and 42 Ma, respectively), regardless of differing rates of relative extinction (Fig. 3). The tribe Heroini (40 Ma) was identified as potentially being exceptionally species rich given clade age based on the estimated background net diversification and relative extinction rates from MEDUSA (Fig. 3B). The remaining four tribes of Central and South American cichlids (Chaetobranchini, Retroculini, Astronotini, and Cichlini) are comparatively depauperate in terms of species diversity, and were not recovered as having exceptional species richness given time, regardless of their divergence time or relative rate of extinction.

## Discussion

This study presents the most globally taxonomically inclusive hypothesis of divergence times across the major lineages of cichlid fishes, and it incorporates representatives from the oldest known fossil cichlids. We recover a Late Cretaceous divergence for the common ancestor of the family Cichlidae, which is consistent with previous Gondwanan vicariance hypotheses that have explained the present distribution of cichlid taxa in Madagascar, India/Sri Lanka, Africa, Iran, and Central and South America (e.g., [23], [24], [46], [47]). Our results also indicate that the common ancestor of each of the monophyletic cichlid subfamilies most likely arose during the Cenozoic (Fig. 1), which is consistent with the known fossil distributions of the oldest described cichlid taxa from these geographic lineages, extending to the Eocene approximately 40 to 49 Ma (e.g. [28], [32], [33]). While Cretaceous-age fossils are currently lacking for the family Cichlidae, a vicariant origin for the family cannot be refuted by the lack of fossils. The East African and Argentinian fossils establish a minimum age for cichlids at ∼40–46 Ma [28], [29], [31], [32], [33] and double the age of cichlids from previously known fossil specimens. Our divergence-time estimates are consistent with both the sequence and timing of Gondwanan breakup, and they indicate that the diversification of cichlid lineages may have occurred in the Mesozoic. The discovery of these older fossil cichlids highlight the possibilities that the fossil record is not complete enough to rule out the future discovery of Cretaceous-aged cichlid fossil; the absence of evidence is not evidence of absence.

Among cichlid subfamilies, the Etroplinae and Ptychochrominae are depauperate with a combined total of 31 valid species [42], accounting for less than two percent of known cichlid diversity. The low species richness in these clades is not caused by a rate shift decrease in net diversification relative to the subfamilies Pseudocrenilabrinae and Cichlinae (Fig. 2). The etroplines and ptychochomines also do not exhibit exceptional species richness given their potential divergence times regardless of the potential relative rate of extinction that may exist in these lineages (Fig. 3). This indicates that the present diversity of the ptychochromines and etroplines may be explained by clade age alone, as these lineages are not so depauperate as to fall outside the lower bound of the expected number of species given their age. Previous studies [24], [34], [48] have suggested that the low diversity of etroplines and ptychochromines may be attributed to limited habitat space and the comparative size of Madagascar and the Indian subcontinent relative to Africa or the Neotropics. In addition, the lack of variable aquatic habitat coupled with the ephemeral nature of many aquatic systems in Madagascar could indicate high extinction rates [48].

An examination of diversification patterns among cichlids with representatives of etropline, ptychochromine, pseudocrenilabrine, and cichline lineages recovered a single net diversification increase within the family. The diversification increase at the African Pseudocrenilabrinae node includes the tribes Oreochromini and Australotilapiini (Fig. 2). The tribe Australotilapiini includes the East African great lake haplochromin cichlids that have long been considered prime examples of adaptive radiations [49], [50], [51]. This tribe also includes the tilapiins and lamprologins, which comprise morphologically diverse assemblages of cichlids, some of which are distributed throughout Africa in a variety of habitats outside of the great-lake systems. Australotilapiin taxa were hypothesized to have undergone a diversification rate shift increase and also unambiguously exhibited exceptional species richness given time (Fig. 3), suggesting the species-rich nature of these lineages cannot be explained by clade age alone given that these lineages most likely diverged relatively recently in the Miocene (Fig. 2, Table 1). A rate-shift increase in this group of cichlids is interesting, but not unexpected given the breadth of literature on great-lake cichlids as potential examples of adaptive radiations [13], [50], [51].

No significant rate shift increases in diversification were detected within the Neotropical subfamily Cichlinae (Fig. 2). Our hypothesis of evolutionary relationships for Cichlinae included a robust sampling of all seven tribes and representative lineages for all Neotropical cichlid tribes. The clade comprising Heroini, Cichlasomatini, Chaetobranchini, and Geophagini encompass the vast majority of Neotropical cichlid diversity; however, only the heroins were found to potentially have elevated rates of diversification relative to other Neotropical cichlid taxa (Fig. 3B), suggesting that clade age alone may not explain the species richness of heroin cichlids. The lack of a significant rate shift in diversification rate in Neotropical lineages (Fig. 2) provides empirical evidence that contradicts previous claims that certain Neotropical lineages may have evolved at significantly elevated rates, such as the geophagins [20], [21], [22], which we find did not diversify at a more rapid rate than the background rate for cichlids nor relative to other Neotropical clades (Figs. 2, 3). Our results indicate that, other than heroins, the species richness in these Neotropical lineages can be explained simply by clade age alone, as the cichlasomatins and the geophagins are shown to lack exceptional species richness given potential clade age and relative extinction rates. Previous work by López-Fernández et al. [22] used lineage through time plots to indicate density-dependent patterns of diversification for Neotropical lineages; however, in our analyses that incorporate knowledge from the known valid described species [42] to account for incomplete taxonomic sampling, we identify no Neotropical cichlid lineages that have undergone a burst in diversification relative to other cichlids (Fig. 2). Our analysis indicates that only the heroins were found to show that their present day species richness may not be explained by clade age alone (Fig. 3B). This is the first study to empirically illustrate that Neotropical cichlids have not undergone any rapid bursts in rates of diversification.

## Conclusions

Our results empirically illustrate that a rate-shift increase in diversification played a prominent role in the evolution of African pseudocrenilabrine lineages, but less so with the Neotropical cichlid lineages. Any number of factors such as habitat availability, competition, or selection could have lead to this rate increase in African cichlids. Interestingly, other lineages of African fishes do not appear to exhibit rate shifts in diversification (e.g. *Synodontis* catfishes) [52]. The absence of a rate shift increase in the diversification rate of Neotropical cichlids (Cichlinae), which comprise nearly one-third of all cichlid diversity, had not previously been corroborated by empirical data, although rapid-diversification among some Neotropical lineages (e.g., geophagins) has previously been hypothesized [20], [21], [22]. Among all Neotropical lineages, only the heroins were identified as having a species richness that may not be simply explained by clade age alone, suggesting that further work is needed to study the macroevolutionary processes that have shaped the evolutionary history of heroin cichlids. These findings aid in our understanding of the diversification patterns across taxonomically disparate lineages in one of the largest clades of freshwater fishes, and one of the most species-rich families of vertebrates.

## Supporting Information

Figure S1 **Strict consensus of seven most parsimonious trees (16549 steps, CI: 0.30, RI: 0.35) resolved for the 54-taxon cichline phylogeny that includes all 51 extant terminals, †** ***Plesioheros,*** **and both species of †** ***Tremembichthys*** **.**
(PDF)

Figure S2 **Single most parsimonious tree (15644 steps, CI: 0.30, RI: 0.35) resolved for the 51-taxon cichline phylogeny that includes just the extant terminals.** Branch lengths represent parsimony changes.
(PDF)

Table S1 **The dataset used in this study for phylogenetic reconstruction included two mitochondrial (large ribosomal subunit 16S, COI) and two nuclear (histone H3, Tmo-4C4) genes for a total of 2,069 aligned nucleotides.**
(PDF)

Table S2 **List of cichlid taxa used in supplemental cichlid phylogenetic analysis.**
(PDF)

Methods S1 **Detailed methods for fossil calibrations and cichlid taxonomic estimates for diversification analyses.**
(DOC)
