# Supplementary figures and images for: Temporal Patterns of Diversification across Global Cichlid Biodiversity (Acanthomorpha: Cichlidae)
Source: PLoS One. 2013 Aug 19;8(8):e71162. doi: 10.1371/journal.pone.0071162 (PMC3747193; doi:10.1371/journal.pone.0071162)

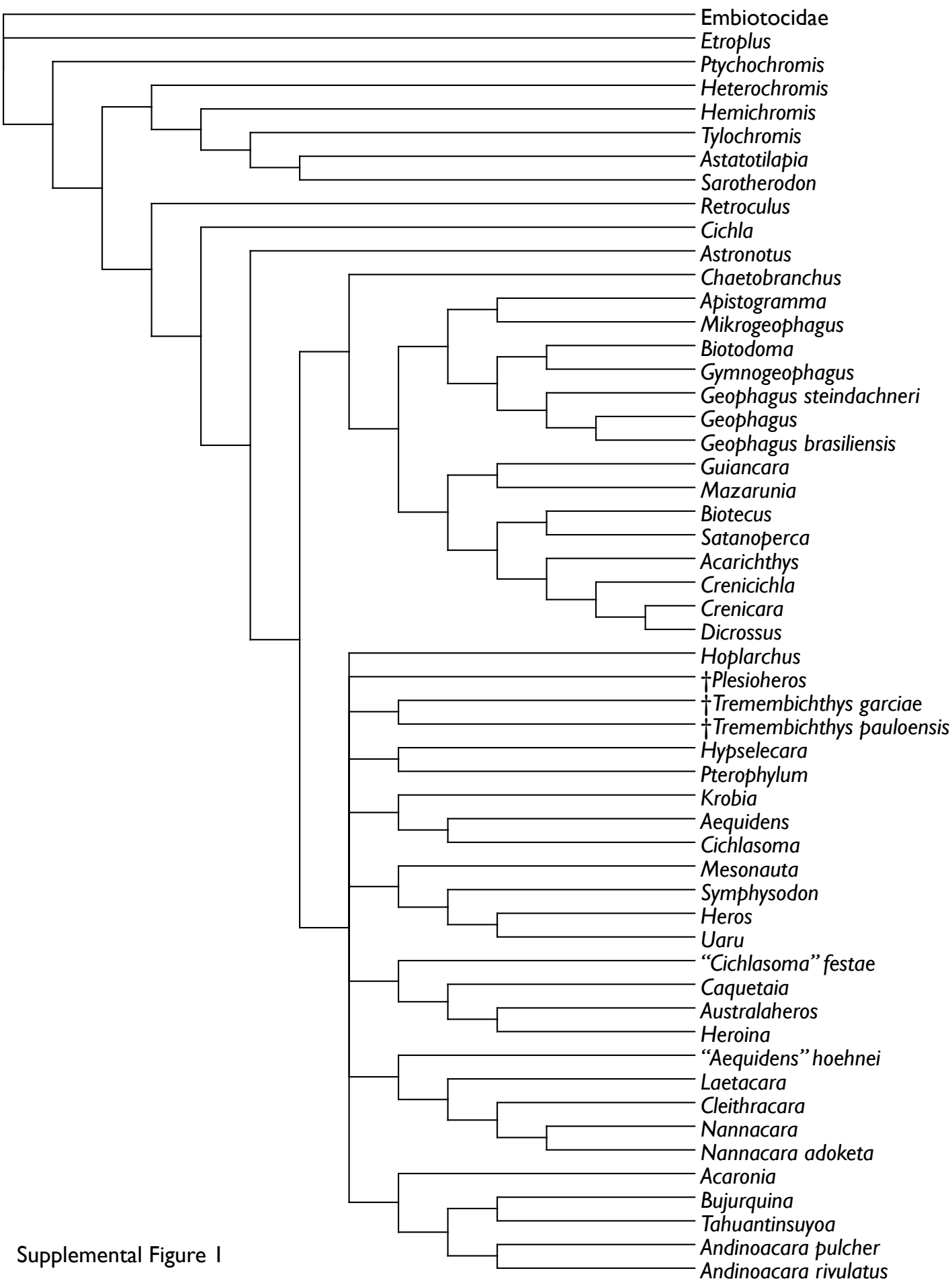

Supplemental Figure I

Supplement: Figure S1 — Strict consensus of seven most parsimonious trees (16549 steps, CI: 0.30, RI: 0.35) resolved for the 54-taxon cichline phylogeny that includes all 51 extant terminals, † Plesioheros, and both species of † Tremembichthys . (PDF) [file pone.0071162.s001.pdf]

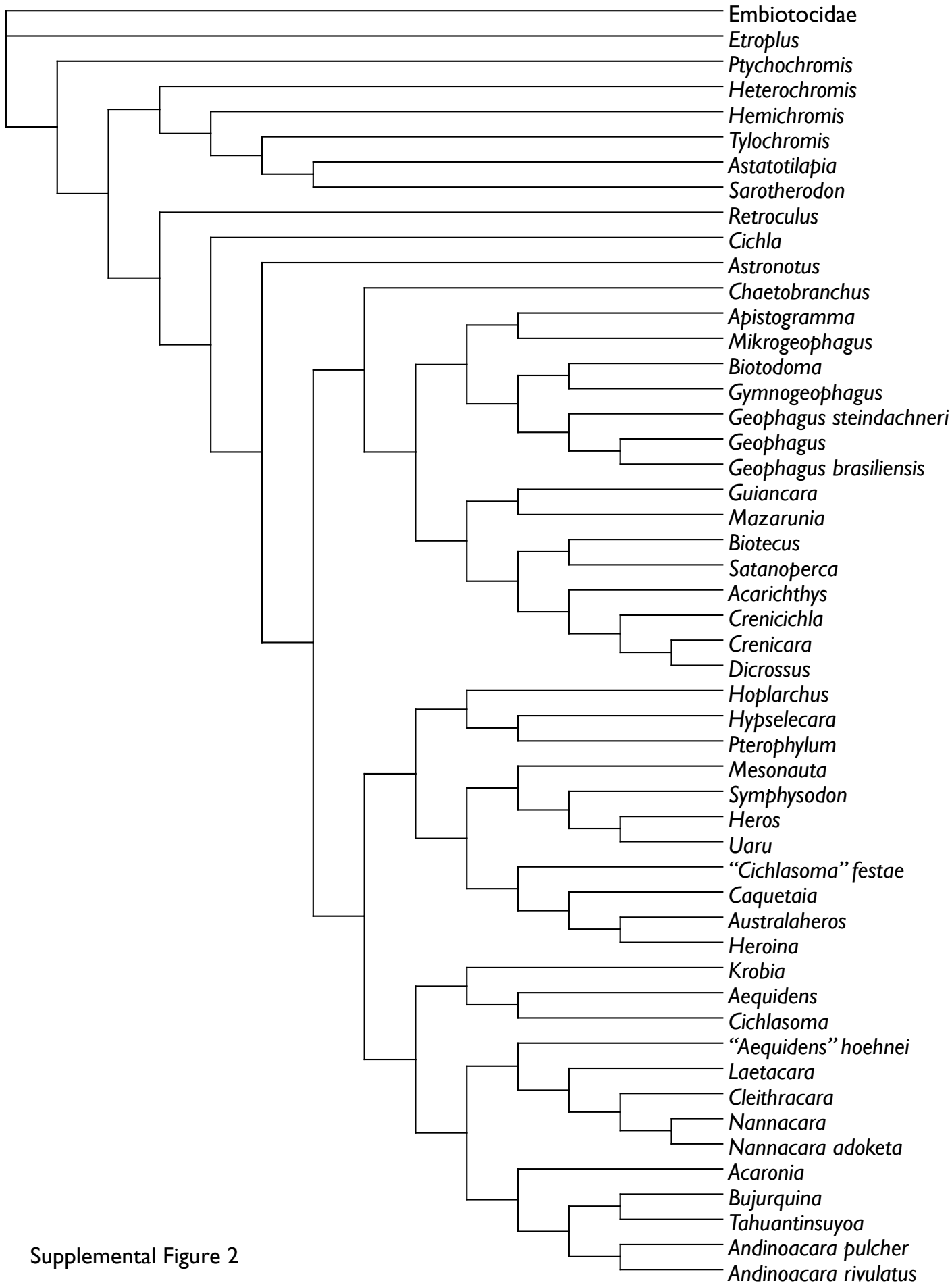

Supplemental Figure 2

Supplement: Figure S2 — Single most parsimonious tree (15644 steps, CI: 0.30, RI: 0.35) resolved for the 51-taxon cichline phylogeny that includes just the extant terminals. Branch lengths represent parsimony changes. (PDF) [file pone.0071162.s002.pdf]
