# Supplementary material for: Temporal Patterns of Diversification across Global Cichlid Biodiversity (Acanthomorpha: Cichlidae)
Source: PLoS One. 2013 Aug 19;8(8):e71162. doi: 10.1371/journal.pone.0071162 (PMC3747193; doi:10.1371/journal.pone.0071162)
Supplement: Table S1 — The dataset used in this study for phylogenetic reconstruction included two mitochondrial (large ribosomal subunit 16S, COI) and two nuclear (histone H3, Tmo-4C4) genes for a total of 2,069 aligned nucleotides. (PDF) [file pone.0071162.s003.pdf]

Supplemental Table 1: Taxa sampled for the phylogenetic analysis and corresponding GenBank accession numbers.

Terminal Analyzed	16S	COI	4C4	H3
Polymixia lowei	AY538966	AY662744	AY539382	AY539175
Hoplostethus mediterraneus	AY538968	AY662745	AY539384	AY539177
Abudefduf saxatilis	AF285942	AY662765	AY662815	N/A
Amblyphidodon leucogaster	N/A	AY662764	AY662813	N/A
Amphiprion polymnus	AY666170	N/A	AY662814	AY662889
Badis badis	AY662699	AY662746	AY662799	AY662875
Calamus penna	AY662700	AY662747	AY662800	AY662876
Calloplesiops altivelis	AY662701	AY662748	AY662801	AY662877
Cetoscarus bicolor	AY662707	AY662758	AY662807	AY662883
Ctenopoma acutirostre	AY662702	AY662749	AY662802	AY662878
Cymatogaster aggregata	AY662711	AY662762	AY662811	AY662887
Diplectrum formosum	AY539048	AY662750	AY539456	AY539257
Embiotoca jacksoni	AY662712	AY662763	AY662812	AY662888
Gramma loreto	AY539053	AY662751	AY539461	AY539268
Haemulon plumieri	AY539057	AY662752	A539465	AY539266
Haletta semifasciata	AY662708	AY662759	AY662808	AY662884
Hermosilla azurea	AY662703	N/A	AY662803	AY662879
Lachnolaimus maximus	AY662709	AY662760	AY662809	AY662885
Micrometrus minimus	EU888021	N/A	U70346	EU888022
Monocirrhus polyacanthus	A662704	AY662753	AY662804	AY662880
Morone saxatilis	AY538941	AY662754	AY539454	AY539255
Perca flavescens	AY539055	AY662755	AY539463	AY539264
Polycentropsis abbreviata	AY662705	AY662756	AY662805	AY662881
Pristolepis fasciata	AY662706	AY662757	AY662806	AY662882
Tautoga onitis	AY662710	AY662761	AY662810	AY662886
**Family Cichlidae**				
**Subfamily Etroplinae**				
Etroplus canarensis	AY662713	AY662766	AY662816	AY662890
Etroplus maculatus	AY263830	AY263858	AY662818	AY662892
Etroplus suratensis	AY263829	AY263870	AY662817	AY662891
Paretroplus dambabe	AY263822	AY263851	AY662819	AY662893
Paretroplus damii	AY263827	AY263856	AY662820	AY662824
Paretroplus gymopreopercularis	AY263825	AY263855	AY662822	AY662897
Paretroplus kieneri (Kinkony)	AY263827	AY263854	AY662821	AY662895
Paretroplus kieneri (Betsiboka)	AY662715	AY662768	AY662823	AY662898
Paretroplus lamenabe	AY662717	AY662770	AY662830	AY662905
Paretroplus maculatus	AY263820	AY263872	AY662824	AY662899
Paretroplus maromandia	AY263821	AY263852	AY662825	AY662900
Paretroplus menarambo	AY263823	AY263853	AY662826	AY662901
Paretroplus nourissati	AY263828	AY263857	AY662827	AY662902
Paretroplus polyactis	AY263826	AY263871	AY662828	AY662903
Paretroplus cf. polyactis	AY662718	AY662771	AY662831	AY662906
Paretroplus tsimoly	AY662716	AY662769	AY662829	AY662904
**Subfamily Ptychochrominae**				
Katria katria	AY263814	AY263880	AY662840	AY662915
Oxylapia polli	AY263817	AY263881	AY662832	AY662907
Paratilapia cf. bleekeri	AY263819	AY263885	AY662833	AY662908
Paratilapia polleni	AY662720	AY662772	AY662835	AY662910
Paratilapia sp. (East)	AY263818	AY263884	AY662836	AY662911
Paratilapia sp. (Nosy Be)	AY662719	AY263886	AY662834	AY662909
Paratilapia sp. (Ifasy)	AY662721	AY662773	AY662837	AY662912
Ptychochromis grandidieri	AY263811	AY263878	AY662841	AY662916
Ptychochromis inornatus	AY263812	AY263875	AY662842	AY662917
Ptychochromis insolitus	AY662725	AY662777	AY662847	AY662922
Ptychochromis oligacanthus (Nosy Be)	AY263813	AY263873	AY662843	AY662918
Ptychochromis oligacanthus (Northwest)	AY662722	AY662774	AY662844	AY662919
Ptychochromis makira	AY662724	AY662775	AY662846	AY662922
Ptychochromis loisellei	AY662723	AY662776	AY662845	AY662920
Ptychochromoides betsileanus	AY263815	AY263882	AY662838	AY662913
Ptychochromoides vondrozo	AY263816	AY263883	AY662839	AY662914
**Subfamily Pseudocrenilabrinae**				
Heterochromis multidens	AF048996	EU888027	AF113060	EU888028
Astatoreochromis alluaudi	AY263846	AY662788	AY662859	AY662938
Chalinochromis popelini	AY263844	AY263867	AY662860	AY662939
Diplotaxodon sp.	AY263843	AY263866	AY662861	AY662940
Etia nguiti	AY662736	AY662789	AY662862	AY662941
Gobiocichlaethelwynnae	AY662737	AY662790	AY662863	AY662942
Haplochromis burtoni	EU888023	EU888024	EU888025	EU888026
Haplochromis simpsoni	AY263848	AY662791	AY662864	AY662943
Haplochromis sp. (Silver bullet)	AY263847	AY662892	AY662865	AY662944
Hemichromis guttatus	AY662738	AY662793	AY662866	AY662945
Neolamprologus brichardi	AY263845	AY662794	AY662867	AY662946
Oreochromis esculentus	AY662739	AY662795	AY662868	AY662947
Oreochromis mossambicus	AY263841	AY263864	AY662869	AY662948
Pelmatochromis nigrofasciatus	AY662740	N/A	AY662870	AY662949
Pelviachromis pulcher	AY662741	AY662796	A662871	AY662950
Pseudotropheus zebra	AY263842	AY263865	AY662872	AY662951
Sarotherodon lohbergeri	EU888029	EU888030	EU888031	EU888032
Steatocranus tinanti	AY662742	AY662797	AY662873	AY662952
Tylochromis pulcher	AY662743	AY662798	AY662874	AY662953
**Subfamily Cichlinae**				
Acarichthys heckelii	AY662726	AY662778	AY662848	AY662923
Acaronia nassa	AY263835	AY263862	AY662849	AY662924
Aequidens pulcher	AY294128	EU888039	N/A	EU888040
Aequidens rivulatus	EU888041	EU888042	N/A	EU888043
Aequidens tetramerus	EU888036	EU888037	AF113078	EU888038
Amatitlania nigrofasciata	DQ119167	DQ119196	DQ119225	N/A
Amphilophus citrinellus	DQ119169	DQ119198	DQ119227	N/A
Apistogramma sp.	AY662727	AY662779	AY662850	AY662925
Apistogrammoides pucallpaensis	EU888071	EU888072	N/A	EU888073
Archocentrus centrarchus	DQ119162	DQ119163	DQ119164	N/A
Astronotus ocellatus	AY263832	AY263859	AY662851	AY662926
Australoheros facetus	EU888095	EU888096	N/A	EU888097
Biotodoma wavrini	EU888074	EU888075	EU888076	EU888077
Biotoecus dicentrarchus	EU888064	EU888065	N/A	EU888066
Bujurquina vittata	DQ119186	DQ119215	DQ119244	EU888044
Caquetaia spectabilis	EU888098	EU888099	EU888100	EU888101
Chaetobranchopsis orbicularis	AY662728	AY662780	AY662852	AY662927
Chaetobranchus flavescens	EU888033	EU888034	AF113080	EU888035
Cichla temensis	AY662729	AY662781	AY662853	AY662928
Cichlasoma bimaculatum	AY263836	AY263863	AF113075	AY662929
'Cichlasoma' festae	DQ119187	DQ119216	DQ119245	EU888102
Cleithracara maronii	EU888045	EU888046	N/A	EU888047
Crenicara punctulatum	EU888067	EU888068	EU888069	EU888070
Crenicichla alta	AY263837	AY263860	AY662854	AY662930
Dicrossus sp.	AY662730	AY662782	AY662855	AY662931
Geophagus brasiliensis	EU888080	EU888081	EU888082	EU888083
Geophagus megasema	EU888078	N/A	AF113093	EU888079
Geophagus steindachneri	DQ119188	DQ119217	DQ119246	EU888084
Guianacara sp.	EU888061	EU888062	AF113084	EU888063
Gymngeophagus gymnogenys	EU888085	EU888086	EU888087	EU888088
Herichthys carpinitis	DQ119172	DQ119201	DQ119230	N/A
Heros appendiculatus	DQ119189	DQ119218	DQ119247	EU888103
Herotilapia multispinosa	DQ119166	DQ119195	DQ119224	N/A
Hoplarchus psittacus	EU888104	EU888105	EU888106	EU888107
Hypselecara temporalis	DQ119190	DQ119219	DQ119248	EU888108
Hypsophrys nicaraguensis	DQ119173	DQ118202	DQ119231	N/A
Krobia sp.	EU888048	EU888049	N/A	EU888050
Laetacara thayeri	EU888051	EU888052	AF113079	EU888053
Mesonauta festivus	EU888109	EU888110	AF113066	EU888111
Mikrogeophagus altispinosus	EU888089	EU888090	AF113089	EU888091
Nandopsis ramsdeni	AY662731	AY662787	DQ119182	AY662932
Nannacara taenia	EU888054	EU888055	N/A	EU888056
Parachromis managuensis	DQ119174	DQ119203	DQ119232	N/A
Paraneetroplus melanurus	DQ119180	DQ119209	DQ119238	N/A
Petenia splendida	DQ119177	DQ119206	DQ119235	N/A
Pterophyllum scalarae	AY662732	AY662783	AY662856	AY662933
Retroculus xinguensis	AY662733	AY662784	AY662857	AY662934
Rocio octofasciata	DQ119168	DQ119197	DQ119226	N/A
Satanoperca leucosticta	AY263838	AY263861	AY6629351	AY662935
Symphysodon discus	EU888112	EU888113	AF113069	N/A
Taeniacara candidi	EU888092	EU888093	AF113094	EU888094
Tahuantinsuyoa macantzatza	EU888057	EU888058	EU888059	EU888060
Teleocichla sp.	AY662734	AY662785	AY662858	AY662936
Cichlasoma' wesseli	EU888114	EU888115	N/A	N/A
Thorichthys aureus	DQ119178	DQ119207	DQ119236	N/A
Tomocichla asfraci	AY662735	AY662786	DQ119237	AY662937
Uaru amphiacanthoides	DQ119191	DQ119221	DQ119249	EU888116
