# Supplementary material for: Temporal Patterns of Diversification across Global Cichlid Biodiversity (Acanthomorpha: Cichlidae)
Source: PLoS One. 2013 Aug 19;8(8):e71162. doi: 10.1371/journal.pone.0071162 (PMC3747193; doi:10.1371/journal.pone.0071162)
Supplement: Table S2 — List of cichlid taxa used in supplemental cichlid phylogenetic analysis. (PDF) [file pone.0071162.s004.pdf]

Supplemental Table 2. GenBank numbers of species analyzed for Neotropical cichlid fossil as shown in Supplemental Figures 1-2

**Terminal Analyzed**	**ND4**	**Cyt-b**	**16S**	**RAG2**	**S7**	**COI**	**4C4**	**H3**	**M27**
Embiotocidae	N/A	AF159336	EU888021	N/A	N/A	N/A	U70346	EU888022	U63676
Etroplus	N/A	AF370625	AY263830	AY279874	DQ119250	AY263858	AY662892	AY662818	U63672
Ptychochromis	N/A	AF370630	AY662722	AY279873	N/A	AY662774	AY662844	AY662919	N/A
Astatotilapia	N/A	AF015029	EU888023	N/A	N/A	EU888024	EU888025	EU888026	U63659
Hemichromis	GU736996	AF015017	AY662738	N/A	GU736678	AY662793	AY662866	AY662945	N/A
Heterochromis	GU736993	AF370636	AF948996	GU736811	GU736676	EU888027	AF113060	EU888028	AF112598
Sarotherodon	N/A	AJ844960	EU888029	N/A	N/A	EU888030	EU888031	EU888032	N/A
Tylochromis	N/A	AF370639	AY662743	N/A	N/A	AY662798	AY662874	AY662953	U63656
Acarichthys	AY566768	AF370653	AY662726	AY566733	GU736686	AY662778	AY662848	AY662923	AF112621
Acaronia	GU737029	AF370666	AY263835	GU736845	EF432989	AY263862	AY662849	AY662924	AF112614
Aequidens (sensu stricto)	GU737016	AY050609	EU888036	GU736829	EF432971	EU888037	AF113078	EU888038	AF112616
"Aequidens" hoehnei	N/A	N/A	N/A	N/A	N/A	N/A	N/A	N/A	N/A
Andinoacara pulcher	GU737021	EF432943	AY294128	GU736834	EF432979	EU888039	N/A	EU888040	N/A
Andinoacara rivulatus	GU737020	EF432935	EU888041	GU736833	EF432977	EU888042	N/A	EU888043	N/A
Apistogramma	AY566787	AF370656	AY662727	AY566749	DQ119272	AY662779	AY662850	AY662925	AF112633
Astronotus	AY566776	AB018987	AY263832	AY566740	GU736683	AY263859	AY662851	AY662926	U63668
Australoheros	GU737032	AY998666	EU888095	GU736848	GU736752	EU888096	N/A	EU888097	N/A
Biotodoma	AY566784	AF370657	EU888074	AY566726	GU736693	EU888075	EU888076	EU888077	AF112620
Biotoecus	AY566792	GU736929	EU888064	AY566754	GU736694	EU888065	N/A	EU888066	N/A
Bujurquina	N/A	AF370668	DQ119186	GU736835	DQ119273	DQ119215	DQ119244	EU888044	AF112615
Caquetaia	GU737070	AF370671	EU888098	GU736885	DQ836805	EU888099	EU888100	EU888101	AF112609
Chaetobranchus	GU736998	AF370652	EU8880333	GU736812	GU736684	EU888034	AF113080	EU888035	AF112618
Cichla	AY566793	AF370644	AY662729	AY566755	GU736680	AY662781	AY662853	AY662928	U63666
Cichlasoma (sensu stricto)	AY566778	AF145128	AY263836	AY566747	EF432966	AY263863	AF113075	AY662929	AF112613
"Cichlasoma" festae	GU737031	AY050610	DQ119187	GU736847	DQ836812	DQ119216	DQ119245	EU888044	N/A
Cleithracara	GU737024	AY050614	EU888045	GU736839	EF432993	EU888046	N/A	EU888047	N/A
Crenicara	N/A	AF370655	EU888067	AY566742	GU736695	EU888068	EU888069	EU888070	AF112628
Crenicichla	AY566785	AF370646	AY263837	AY566750	GU736697	AY263860	AY662854	AY662930	AF112625
Dicrossus	AY566767	GU736938	AY662730	AY566731	GU736704	AY662782	AY662855	AY662931	N/A
Geophagus (sensu stricto)	AY566763	AF370658	EU888078	AY566727	GU736706	N/A	AF113093	EU888079	AF112631
Geophagus brasiliensis	AY566766	AF370659	EU888080	AY566732	GU736713	EU888081	EU888082	EU888083	AF112626
Geophagus steindachneri	AY566765	AF370660	DQ119188	AY566730	DQ119275	DQ119217	DQ119246	EU888084	N/A
Guianacara	AY566762	AF370654	EU888061	AY566730	GU736715	EU888062	AF113084	EU888063	AF112622
Gymnogeophagus	AY566775	AF370661	EU888085	AY566738	GU736718	EU888086	EU888087	EU888088	AF112623
Heroina	GU737044	AY998670	GU737198	GU736860	N/A	N/A	N/A	N/A	N/A
Heros	GU737034	DQ010102	DQ119189	GU736849	DQ119276	DQ119218	DQ119247	EU888103	AF112605
Hoplarchus	AY566789	AF370673	EU888104	AY566760	GU736757	EU888105	EU888106	EU888107	AF112612
Hypselecara	GU737037	AY050612	DQ119190	GU736853	DQ119277	DQ119219	DQ119248	EU888108	AF112611
Krobia	GU737025	EF432931	EU888048	GU736840	EF432961	EU888049	N/A	EU888050	N/A
Laetacara	GU737026	AY050608	EU888051	GU736842	EF433001	EU888052	AF113079	EU888053	AF112617
Mazarunia	GU737012	GU736960	GU737165	GU736825	GU736729	N/A	N/A	N/A	N/A
Mesonauta	AY566782	DQ494392	EU888109	AY566748	DQ836809	EU888110	AF113066	EU888111	AF112604
Mikrogeophagus	AY566764	GU736953	EU888089	AY566729	N/A	EU888090	AF113089	EU888091	AF112627
Nannacara (Ivanacara)	N/A	EF432946	EF432903	N/A	EF432995	N/A	N/A	N/A	N/A
Nannacara (sensu stricto)	N/A	EF432921	EU888054	GU736843	EF432991	EU888055	N/A	EU888056	N/A
Pterophyllum	GU737039	AF370676	AY662732	GU736855	GU736761	N/A	AY662856	AY662933	AF112603
Retroculus	AY566774	AF370641	AY662733	AY566737	GU736685	AY662784	AY662857	AY662934	AF112600
Satanoperca	AY566783	AB018986	AY263838	AY566745	GU736723	AY263861	AY6629351	AY662935	N/A
Symphysodon	GU737041	AY840119	EU888112	GU736857	GU736763	EU888113	AF113069	N/A	AF112607
Tahuantinsuyoa	GU737028	EF432915	EU888057	GU736844	EF432983	EU888058	EU888059	EU888060	N/A
Uaru	GU737042	AF370678	DQ119191	GU736859	DQ119278	DQ119221	DQ119249	EU888116	AF112606
