# Supplementary material for: Temporal Patterns of Diversification across Global Cichlid Biodiversity (Acanthomorpha: Cichlidae)
Source: PLoS One. 2013 Aug 19;8(8):e71162. doi: 10.1371/journal.pone.0071162 (PMC3747193; doi:10.1371/journal.pone.0071162)
Supplement: Methods S1 — Detailed methods for fossil calibrations and cichlid taxonomic estimates for diversification analyses. (DOC) [file pone.0071162.s005.doc]

**Additional Materials**

Fossil Calibrations

Fossil calibrations. All fossil calibrations were assigned a lognormal prior, with hard minimum ages of clades set *a priori*. The minimum dates were assigned based on the oldest known fossil of each clade discussed below. The calibration points are noted on Figure 1.

Acanthomorpha (C1): The node representing the MRCA (most recent common ancestor) of Acanthomorpha was given a minimum age of 95 Ma (million years ago), based on the fossil taxon †*Polymixia* sp., known from Middle­–Upper Cenomanian deposits [1]. A conservative soft upper bound was set to 150 Ma, the age of the oldest known fossil euteleost †*Leptolepides sprattiformis* [2, 3]. The lognormal prior was given an offset of 95 Ma, with a standard deviation of 1.0 and a mean of 2.0.

Cichlidae – Pseudocrenilabrinae less *Heterochromis* (C2): A minimum age of 46 Ma was assigned based on the African cichlid, **†***Mahengechromis* sp., from Eocene deposits [4, 5]. A conservative soft upper bound was set to 94 Ma, the age of the above “trachichthyoids” **†***Hoploteryx lewesiensis* and **†***H. simus* [1]. The lognormal prior was given an offset of 46 Ma, with a standard deviation of 1.0 and a mean of 1.95.

Cichlidae – *Geophagus* + *Gymnogeophagus* (C3): A minimum age of 40 Ma was assigned to the MRCA of the clade *Geophagus* + *Gymnogeophagus* based on the phylogenetic position of †*Gymnogeophagus eocenicus*, from the Eocene Lumbrera Formation in northwestern Argentina [6]. A soft upper bound of 95 Ma was set on the lognormal prior (offset of 40, standard deviation of 1.0, mean of 1.88).

Cichlidae – Cichlasomatini+Heroini (C4): A minimum age of 40 Ma was assigned based on Neotropical cichlids, **†***Plesioheros* and **†***Tremembichthys*, from Eocene deposits [7-9]. A conservative soft upper bound was set to 94 Ma, the age of the above “trachichthyoids” **†***Hoploteryx lewesiensis* and **†***H. simus* [1]. The lognormal prior was given an offset of 40 Ma, with a standard deviation of 1.0 and a mean of 1.88.

South American Cichlid Phylogenetics for Fossil Calibrations of †*Plesioheros* and †*Tremembichthys*

*Taxon Sampling.*­­­—A total of 54 taxa were analyzed in this supplemental study (Supporting Table S2). The topology was rooted with the surfperch, Embiotocidae, and it included one Indian (*Etroplus*), one Malagasy (*Ptychochromis)*, and five African cichlid terminals (*Heterochromis, Hemichromis, Tylochromis, Astatotilapia,* and *Sarotherodon*). The remaining 46 species are either extant generic sampling (43 spp.) or fossil (three spp.) species that had been previously coded in morphological phylogenetic analyses [8-10]. Because our emphasis was on the placement of the fossil taxa for calibration points, we only included extant taxa from Kullander [11] and Smith et al. [12] that included morphological data. Central American genera analyzed by Smith et al. [12] without morphological data, for example, were excluded from the analysis.

*Character Sampling.*—A total of 91 morphological features and 5,945 nucleotide characters were analyzed from four mitochondrial loci (16S, COI, Cyt-*b*, and ND4) and five nuclear loci (Histone H3, RAG-2, S7, TMO-4C4, and TMO-M27). The 51 extant genus-level terminals with molecular data analyzed in the present study and the gene fragments sequenced are listed in Supporting Table S2. Most of these sequences were utilized in Smith et al. [12], but new ND4, RAG-2, and S7 sequences that were subsequently published in López-Fernández et al. [13] were added to the analysis (Supporting Table S2). These molecular data were simultaneously analyzed with a morphological dataset composed of 91 characters that was based on Malabarba and Malabarba [8] for **†***Tremembichthys*, Smith et al. [12], and Perez et al. [9] for **†***Plesioheros*. Smith et al. [12] was previously based on Kullander [11, 14] and Malabarba et al. [10].

*Phylogenetic analyses.*— The phylogenetic analysis was an updated analysis using the same methods and search strategy as published by Smith et al. [12]. To assess the impacts of missing data in the fossils on the final topology, we performed one analysis with the fossils removed.

*Results.*— We show a 54-taxon analysis that included †*Plesioheros,* both species of †*Tremembichthys,* and all 51 extant species (Supporting Figure S1) and a 51-taxon analysis that excluded all three fossils (Supporting Figure S2). Generally speaking, the fossils reduced resolution in the trees, but did not, otherwise, substantially alter the topology (Supporting Figure S1) relative to the analysis without fossils (Supporting Figure S2). Based on the results of these analyses, we were able to provide an up-to-date placement of the cichline fossils.

The non-fossil changes to the relationships presented herein compared to Smith et al. [12] are minimal: *Retroculus* moved to the base of the Cichlinae (rather than sister to *Cichla*), *Pterophylym* moved to a more basal placement in the Heroini, *Australoheros* and *Symphysanodon* moved to more apical positions within Heroini, *Satanoperca* moved to the crenicaratins from the geophaginins, *Mazarunia* moved from the geophaginins to the acarichthyins, and *Geophagus steindachneri* moved to a more basal placement within Geophagina. For more discussion of cichline relationships, see Smith et al. [12] or López-Fernández et al. [13]. These revised phylogenetic hypotheses allow us to place minimal ages for multiple nodes within cichlids for the BEAST analysis presented in Figure 1. Our results indicate that both **†***Plesioheros* and **†***Tremembichthys* are resolved at the base of the clade composed of the Cichlasomatini and Heroini (Supporting Figure S1).

**Cichlid Taxonomic Estimates for Diversification Analyses**

Taxonomic estimates with one representative from each tribe and or subfamily as a terminal for combined taxonomic and phylogenetic analyses are described below (Fig. 2). Taxonomic estimates are based on the number of valid described and diagnosed species from the Catalog of Fishes [15], and include 16 species of subfamily Etroplinae from Madagascar and India, and 15 species of subfamily Ptychochrominae from Madagascar. Estimates for the African subfamily Pseudocrenilabrinae were reduced to tribes that include 1 species of Heterochromini, 4 species of Pelmatochromini, 18 species of Tylochromini, 12 species of Hemichromini, 51 species of Chromidotilapini, 1 species of *Etia*, 36 species of Boreochromini, 75 species of Oreochromini, and 883 species of Australotilapiini. In particular, the tilapiine cichlids have been shown to be paraphyletic [16]. For this study we follow Schwarzer et al. [16] as close as possible for taxonomic assignment of tilapiine cichlids. Schwarzer et al. [16] recovered a clade (Oreochromini) comprising the tilapiine genera *Sarotherodon*, *Oreochromis*, *Alcolapia*, *Tristamella*, *Iranocichla*, and *Stomatepia*. This corresponds to our Tilapiini A clade. The Boreochromini clade of Schwarzer et al. [16] is represented in our tree by the genus *Gobiocichla*. Finally, we recover a clade corresponding to the Australotilapiini clade of Schwarzer et al. [16]. Based on previous phylogenetic studies of Pseudocrenilabrinae [12, 16, 17], we believe this is the most logical scenario at present for assigning taxonomic diversity to pseudocrenilabrid lineages, pending future large-scale phylogenetic studies of the Pseudocrenilabrinae. Tribe estimates for the South and Central American subfamily Cichlinae include; 238 species of Geophagini, 115 species of Cichlasomatini, 148 species of Heroini, 15 species of Cichlini, 5 species of Chaetobranchini, 3 species of Retroculini, and 2 species of Astronotini.

**References**

1. Patterson C (1993) Osteichthys: Teleostei. In Benton MJ, editor. The Fossil Record 2. pp. 621-656.
2. Arratia G (1997) Basal teleosts and teleostean phylogeny. Palaeo Ichthyologica 2: 5-168.
3. Arratia G (1999) Systematics and Fossil Record. In Arratia G, Schultze H-P, Verlag Mesozoic Fishes 2. Verlag, Germany. pp. 265-334.
4. Murray, AM (2000) Eocene cichlid fishes from Tanzania, East Africa. J. Vert. Paleontol. 20: 651-664.
5. Murray, AM (2001) The oldest fossil cichlids (Teleostei: Perciformes): indication of a 45 million-year-old species flock. Proc. Roy. Soc., B. 269: 679-684.
6. Malabarba MC, Malabarba LR, Del Papa C (2010) *Gymnogeophagus eocenicus*, n. sp. (Perciformes: Cichlidae), an Eocene cichlid from the Lumbrera Formation in Argentina. J Vert Paleontol 30(2): 341-350.
7. Del Papa, CE (2006) Estratigrafía y paleoambientes de la Formación Lumbrera, Grupo Salta, Noroeste Argentino. Rev. Asoc. Geol. Argentina 61: 313-327.
8. Malabarba, MC, Malabarba LR (2008) A new cichlid *Tremembichthys garciae* (Actinopterygii, Perciformes) from the Eocene-Oligocene of Eastern Brazil. Rev. Bras. Paleontol. 11: 59-68.
9. Perez, PA, Malabarba MC, Del Papa C (2010) A new genus and species of Heroini (Perciformes: Cichlidae) from the early Eocene of southern South America. Neotrop. Ichthy. 8: 631-642.
10. Malabarba MC, Zuleta O, Del Papa C (2006) *Proterocara argentina*, a new fossil cichlid from the Lumbrera formation, Eocene of Argentina. J. Vert. Paleont. 26: 267-275.
11. Kullander SO (2003) Family Cichlidae. In: Reis RE, Kullander SO, Ferraris CJ, editors. Checklist of the Freshwater Fishes of South and Central America. EDIPUCRS, Porto Alegre. pp. 605-654.
12. Smith WL, Chakrabarty P, Sparks JS (2008) Phylogeny, taxonomy, and evolution of neotropical cichlids (Teleostei: Cichlidae: Cichlinae). Cladistics 24: 625-641.
13. López-Fernández H, Honeycutt RL, Winemiller KO (2010) Multilocus phylogeny and rapid radiations in Neotropical cichlid fishes (Perciformes: Cichlidae: Cichlinae). Mol Phylogenet Evol 55: 1070-1086.
14. Kullander SO (1990) *Mazarunia mazarunii* (Teleostei: Cichlidae), a new genus and species from Guyana, South America. Ichthyol. Explor. Freshwaters 1: 3-14.
15. Eschmeyer WN (2012) Catalog of Fishes. California Academy of Sciences (http://research.calacademy.org/research/ichthyology/catalog/fishcatmain.asp). Electronic version accessed 19 November 2012.
16. Schwarzer J, Misof B, Tautz D, Schliewen UK (2009) The root of the East African cichlid radiations. BMC Evol Biol 9: 186. doi: 10.1186/1471-2148-9-186.
17. Klett V, Meyer A (2002) What, if anything, is a tilapia? Mitochondrial ND2 phylogeny of Tilapiines and the evolution of parental care systems in the African cichlid fishes. Mol Biol Evol 19(6): 865-883.

**Figure Legend**

Supporting Figure S1 – Strict consensus of seven most parsimonious trees (16549 steps, CI: 0.30, RI: 0.35) resolved for the 54-taxon cichline phylogeny that includes all 51 extant terminals, **†***Plesioheros,* and both species of **†***Tremembichthys.*

Supporting Figure S2 – Single most parsimonious tree (15644 steps, CI: 0.30, RI: 0.35) resolved for the 51-taxon cichline phylogeny that includes just the extant terminals. Branch lengths represent parsimony changes.
